# The Oxidative and Inflammatory State in Patients with Acute Renal Graft Dysfunction Treated with Tacrolimus

**DOI:** 10.1155/2016/5405847

**Published:** 2016-10-30

**Authors:** Sandra Carrillo-Ibarra, José Ignacio Cerrillos-Gutiérrez, Ariadna Escalante-Núñez, Enrique Rojas-Campos, Benjamín Gómez-Navarro, Sonia Sifuentes-Franco, Ernesto Germán Cardona-Muñoz, Alejandra Guillermina Miranda-Díaz

**Affiliations:** ^1^Institute of Experimental and Clinical Therapeutics, Department of Physiology, University Health Sciences Centre, University of Guadalajara, Guadalajara, JAL, Mexico; ^2^Department of Nephrology and Transplants, Specialties Hospital, National Occidental Medical Centre, The Mexican Social Security Institute, Guadalajara, JAL, Mexico; ^3^Kidney Diseases Medical Research Unit, Specialties Hospital, National Occidental Medical Centre, The Mexican Social Security Institute, Guadalajara, JAL, Mexico

## Abstract

*Objective.* To determine the oxidative stress/inflammation behavior in patients with/without acute graft dysfunction (AGD) with Tacrolimus.* Methods.* Cross-sectional study, in renal transplant (RT) recipients (1-yr follow-up). Patients with AGD and without AGD were included. Serum IL-6, TNF-*α*, 8-isoprostanes (8-IP), and Nitric Oxide (NO) were determined by ELISA; C-reactive protein (CRP) was determined by nephelometry; lipid peroxidation products (LPO) and superoxide dismutase (SOD) were determined by colorimetry.* Results.* The AGD presentation was at 5.09 ± 3.07 versus 8.27 ± 3.78 months (*p* < 0.001); CRP >3.19 mg/L was found in 21 versus 19 in the N-AGD group (*p* = 0.83); TNF-*α* 145.53 ± 18.87 pg/mL versus 125.54 ± 15.92 pg/mL in N-AGD (*p* = 0.64); IL-6 2110.69 ± 350.97 pg/mL versus 1933.42 ± 235.38 pg/mL in N-AGD (*p* = 0.13). The LPO were higher in AGD (*p* = 0.014): 4.10 ± 0.69 *µ*M versus 2.41 ± 0.29 *µ*M; also levels of 8-IP were higher in AGD 27.47 ± 9.28 pg/mL versus 8.64 ± 1.54 pg/mL (*p* = 0.01). Serum levels of NO in AGD were lower 138.44 ± 19.20 *µ*mol/L versus 190.57 ± 22.04 *µ*mol/L in N-AGD (*p* = 0.042); antioxidant enzyme SOD activity was significantly diminished in AGD with 9.75 ± 0.52 U/mL versus 11.69 ± 0.55 U/mL in N-AGD (*p* = 0.012).* Discussion.* Patients with RT present with a similar state of the proinflammatory cytokines whether or not they have AGD. The patients with AGD showed deregulation of the oxidative state with increased LPO and 8-IP and decreased NO and SOD.

## 1. Introduction

Chronic end-stage renal disease (ESRD) is characterized by serious, irreversible kidney damage distinguished by proteinuria and a reduction in the glomerular filtration rate (GFR) [1, 2].

According to the Registry of Dialysis and Transplants in the State of Jalisco (*Registro de Diálisis y Trasplante del Estado de Jalisco: REDTJAL*) and in the State of Morelos, the number of new patients who require renal replacement therapy (RRT) is constantly increasing [1, 2]. The inherent annual cost reported by the Ministry of Health in Mexico per patient with ESRD in 2012 was $8,966.00 USD while at the Mexican Social Security Institute (*Instituto Mexicano del Seguro Social: IMSS*) it was $9,091.00 USD [3]. The options available for the management of ESRD are peritoneal dialysis (PD), hemodialysis (HD), and renal transplantation (RT) [4]. Renal transplantation is considered the best therapeutic option for patients with ESRD [5]. The United States Renal Data System (USRDS) reports that the incidence rate of RT in Jalisco is among the highest in the world, based on the latest USRDS report where Taiwan, Jalisco (Mexico), and the United States are indicated as the highest incidence of treated ESRD (458, 421, and 363 per million of population (pmp), resp.) [5].

The introduction of Cyclosporine A (CsA) followed by the rapid succession of Tacrolimus (TAC) together with Mycophenolate Mofetil (MMF) is associated with a much lower rate of loss of the renal allograft, achieving graft survival in >90% annually [6]. The most common and feared adverse effect of TAC is nephrotoxicity [7]. Among the greatest challenges in the management of RT is acute rejection (AR), although with new immunosuppressant strategies the frequency of AR has been reduced in the last decades, with significant long-term improvement in survival of the graft and the patient [8, 9]. In Mexico, survival of the graft and survival of the patient are both comparable to those reported by other countries [10, 11]. However, despite the efficacy of actual immunosuppressive regimes for the prevention of AR, nephrotoxicity and infections can influence the appearance of renal graft dysfunction [12]. Only in transplantation between identical twins could immunosuppressive therapy not be required: all RT recipients require immunosuppression in order to avoid rejection. Therefore, it is fundamental to find a balance between the immunosuppressor effect of the drugs and the immunological response of the host in order to prevent the appearance of opportunistic infections that cause the inflammatory state and acute dysfunction of the graft (AGD) [13]. The inflammatory state plays an important role in the interrelationship with oxidative stress (OS) in ESRD and in RT recipients [14, 15]. The OS is characterized by the imbalance between the generation of the oxidant and antioxidant systems. The primary sources of generation of reactive oxygen species (ROS) after RT are found in ischemia-reperfusion and immunosuppression. The reperfusion injury is a common phenomenon in the transplanted kidney and can cause dysfunction of the allograft during the first posttransplant week [16].

Levels of the proinflammatory cytokines like interleukin 6 (IL-6), the tumor necrosis factor alpha (TNF-*α*), the C-reactive protein (CRP), and the markers of OS have been reported as being significantly elevated in ESRD, with significant decreases two months after RT [17].

The AGD is defined as the slow and progressive deterioration of renal function habitually accompanied by proteinuria of varying degrees and hypertension. The most frequent causes of dysfunction are rejection mediated by antibodies, interstitial fibrosis, and tubular atrophy of unspecific origin. The diagnosis of the cause of AGD requires renal biopsy [18]. Since TAC can cause acute tubule-interstitial nephropathy and because the mechanisms of renal damage associated with immunosuppression are not entirely understood, we proposed the objective of determining the behavior of the markers of oxidative stress and inflammation in patients with AGD and without AGD (N-AGD), treated with TAC.

## 2. Patients and Methods

### 2.1. Study Design

An analytical cross-sectional study was performed. Two study groups were made of RT recipients within the first year of follow-up. The first group included all patients with AGD (elevation of creatinine ≥30%) biopsy proven (all biopsies were evaluated by the same pathologist and the diagnosis was recorded), in the period of Jan-2014 to Dec-2015; control group included patients without AGD (N-AGD); these patients were randomly obtained from the pool of patients who were unto protocolized graft biopsy (this is a common behavior in our setting); they were rejection-free at the time of biopsy. All patients were on triple immunosuppression scheme based on TAC, MMF, and Prednisone (considered as the most potent scheme). Both groups were first-time recipients of RT. The calculation of the sample size was determined using the formula to compare means and considering, as a variable in the determination, concentration of the superoxide dismutase enzyme (SOD), obtaining 55 patients per study group. The patients were attended to at the Department of Nephrology, Transplant Division, of the Sub-Specialties Medical Unit at the National Occidental Medical Centre of the Mexican Social Security Institute (*IMSS* in Spanish). The ingestion of TAC for immunosuppressant therapy was considered an inclusion criterion for both groups. Excluded were the patients >55 years of age, who presented with renal comorbidities, who received a second transplant, and who were undergoing treatment with nonsteroidal anti-inflammatories, angiotensin converting enzyme (ACE) inhibitors, and antagonists of the angiotensin II receptors (ARBs), as well as recipients of transplants from perished donors. The serum levels of IL-6, TNF-*α*, 8-isoprostanes (8-IP), and Nitric Oxide (NO) were determined with ELISA. Nephelometry was used to determine CRP and the colorimetric method was used to determine levels of the products of lipid peroxidation (LPO) (malondialdehyde (MDA) and 4-hydroxy-alkenals) and levels of the SOD enzyme as an antioxidant.

### 2.2. Biochemical Analysis

Once blood samples were collected in two separate tubes (one with 0.1% of ethylenediaminetetraacetic (EDTA) and the other a dry tube), the plasma and serum were separated by centrifugation at 2,000 rpm for 10 minutes at room temperature. Then, the samples were stored at −80°C until processing. All of the technical readings of optical density were made with the Synergy HT (BIOTEK®) microplate reader.

### 2.3. TNF-*α* and IL-6

TNF-*α* levels were determined by ELISA, following the instructions of the kit manufacturer (Peprotech, Rocky Hill, NJ 08553, USA). First, 100 *μ*L of diluted capture antibody was added, followed by incubation overnight at room temperature. Then, 300 *μ*L of block buffer was added to the wells and it was incubated for 1 hour at room temperature. Serum and standards were added, followed by incubation for 2 hours at room temperature. After several washings, 100 *μ*L of diluted detection antibody was added and incubated at room temperature for 2 hours. 100 *μ*L diluted Avidin-HRP conjugate was added, followed by incubation for 30 minutes at room temperature. Finally, 100 *μ*L of substrate solution was added to each well. The plate was read at a wavelength of 405 nm with correction set at 650 nm and was reported in pg/mL.

### 2.4. Human High Sensitivity C-Reactive Protein (hsCRP)

Levels of the hsCRP in serum were assessed with immune nephelometry, using the BN II System (Siemens, USA). The reagents, controls SL/1 and SL/2, CardioPhase hsCRP (polystyrene particles coated with mouse monoclonal antibodies to CRP), and N rheumatology standard were prepared according to the manufacturer's instructions. The reference curve was made, and the samples were diluted 1 : 20 and mixed with the CardioPhase hsCRP. The hsCRP levels were measured automatically by the nephelometry BN II. The levels are reported automatically in mg/L.

### 2.5. Products of Lipid Peroxidation

Plasma LPO levels were measured using the FR22 assay kit (Oxford Biomedical Research Inc., Oxford, MI, USA) according to the manufacturer's instructions. In this assay the chromogenic reagent reacts with MDA and 4-hydroxy-alkenals to form a stable chromophore. First, 140 *μ*L of serum with 455 *μ*L of N-methyl-2-phenylindole in acetonitrile (Reagent 1) was diluted with ferric iron in methanol. Samples were agitated, after which 105 *μ*L 37% HCl was added, followed by incubation at 45°C for 60 minutes and centrifugation at 12,791 rpm for 10 minutes. Next, 150 *μ*L of the supernatant was added and absorbance was measured at 586 nm. The curve pattern with known concentrations of 1,1,3,3-tetramethoxypropane in Tris-HCl was used.

### 2.6. 8-Isoprostane (8-IP) Evaluation

The immunoassay reagent kit from Cayman Chemical Company® (Michigan, USA) was used according to the manufacturer's instructions. The 8-IP assay was based on the principle of competitive binding between sample 8-IP, 8-IP acetyl cholinesterase (AChE) conjugate, and 8-IP tracer. 50 *μ*L of samples or standard was added to each well and 50 *μ*L of 8-IP AChE tracer was added to all wells except the total activity and blank wells. 50 *μ*L of 8-IP enzyme immunoassay antiserum was added to all wells except the total activity and blank wells. At once, 50 *μ*L of 8-IP antiserum was added to all wells except total activity, nonspecific binding, and blank wells. The plate was covered and incubated at 4°C for 18 h and then washed 5 times with buffer. Absorbance was read at 420 nm.

### 2.7. Nitric Oxide (NO)

The levels of NO in serum were assessed by sandwich ELISA using a commercially available kit (Human Total Nitric Oxide ELISA Kit, MyBioSource®, San Diego, CA, USA). Before performing the assay samples, reagents were kept at room temperature for 30 min. Nitric Oxide or serum (50 *μ*L) was pipetted into an antibody-coated 96-well plate with 100 *μ*L of HRP-conjugate reagent and incubated at 37°C for 1 h. The wells were then washed four times with buffer wash; 50 *μ*L of chromogen solution A and 50 *μ*L of chromogen solution B were added. The samples were incubated for 15 minutes at 37°C; then 50 *μ*L of stop solution was added, and the absorbance was read at 450 nm.

### 2.8. Superoxide Dismutase

Serum total SOD activity (U/mL) was determined using a kit from Cayman Chemical Company®, (USA, number 706002), following the manufacturer's protocol, to detect the O^2−^ generated by the xanthine oxidase and hypoxanthine enzymes through the reaction of tetrazolium salts. The serum samples were diluted 1 : 5 in sample buffer, 200 *μ*L of the radicals' detector (diluted 1 : 400) was placed, and 10 *μ*L of the sample was added. After slow agitation, 20 *µ*L of xanthine oxidase was added to the wells. The microplate was incubated for 20 minutes at room temperature, and the absorbency was read at a wavelength of 440 nm. Levels are reported in U/mL.

### 2.9. Ethical Considerations

The study was performed in accordance with the Principles of Ethics for Medical Research in Human Beings as stipulated by the Declaration of Helsinki 64th General Assembly, Fortaleza, Brazil (October 2013). Informed consent forms were signed, because it was a category III study, in agreement with the General Health Law in Mexico. The project was submitted to and approved by the local Scientific Research and Health Ethics Committee of the* IMSS *(Registration number R-2015-1301-83) and the State Research Registry (59/E-JAL/2015) put forth by the General Public Health Administration (*Dirección General de Salud Pública*) in Jalisco, Mexico.

### 2.10. Statistical Analysis

Continuous variables are expressed as mean ± standard deviation (SD) or standard error of the mean (SEM) and were analyzed with nonparametric tests according to the results obtained by the Kolmogorov-Smirnov test. For the comparisons between groups the Mann–Whitney *U* test was used. The categorical variables are presented as frequencies and percentages and were analyzed with the Chi^2^ test. A value of *p* ≤ 0.05 was considered statistically significant.

## 3. Results

### 3.1. Demographic and Metabolic Characteristics

The AGD was developed at 5.09 ± 3.07 after transplant (*p* < 0.001) versus 8.27 ± 3.78 months in N-AGD (this was the time of follow-up for protocol biopsy). The age of patients with AGD was 25.39 ± 5.71 years and 28.08 ± 9.12 years in N-AGD. The male gender significantly predominated in both groups (*p* = 0.004): there were 48 (87%) males in the AGD group and 34 (62%) in the N-AGD group. Heights and weights between the AGD and N-AGD groups were not significantly different. Tobacco use (smoking) was present in 15 patients with AGD and in 8 N-AGD. Alcoholism was present in 14 patients with AGD and in 9 of the N-AGD. Differences in fasting glucose levels were not statistically significant. Findings of uremia were significantly increased in the AGD group with 54.78 ± 3.99 mg/dL (*p* < 0.001) versus the N-AGD with 36.59 ± 1.32 mg/dL, and the same behavior was found in levels of serum creatinine which was significantly higher in AGD with 1.19 ± 0.20 mg/mL (*p* = 0.002) versus 1.06 ± 0.27 mg/mL in N-AGD. The age of the donor was significantly higher in the AGD group with 42.54 ± 11.45 years (*p* < 0.001) versus 34.07 ± 10.65 years in N-AGD. The triglycerides and cholesterols were not significantly different between the groups (Table 1).

### 3.2. Proinflammatory Cytokines

Levels of high sensitivity CRP ≤3.19 mg/L were found in 34 AGD patients and >3.19 mg/L in 21 patients. In the N-AGD recipients, 36 patients had ≤3.19 mg/L and 19 had >3.19 mg/L, without significant differences (*p* = 0.83). The average serum level of TNF-*α* in AGD was 145.53 ± 18.87 pg/mL versus 125.54 ± 15.92 pg/mL in N-AGD, without a significant difference (*p* = 0.64). Serum levels of IL-6 in AGD patients were 2110.69 ± 350.97 pg/mL versus 1933.42 ± 235.38 pg/mL in N-AGD, which was not significantly different (*p* = 0.13) (Table 1).

### 3.3. Markers of Oxidative Stress

Serum levels of LPO in AGD were significantly elevated (*p* = 0.014, Mann–Whitney *U* test) with 4.10 ± 0.69 *μ*M versus 2.41 ± 0.29 *μ*M in N-AGD. A similar behavior was found in 8-IP since the average plasma level in AGD patients was also significantly elevated with 27.47 ± 9.28 pg/mL versus 8.64 ± 1.54 pg/mL in N-AGD (*p* = 0.01, Mann–Whitney *U* test).

However, it is noteworthy that the serum levels of NO in the AGD group were found to be significantly diminished with 138.44 ± 19.20 *μ*mol/L versus 190.57 ± 22.04 *μ*mol/L in N-AGD (*p* = 0.042, Mann–Whitney *U* test). The activity of the SOD enzyme was significantly diminished in the AGD group with 9.75 ± 0.52 U/mL versus 11.69 ± 0.55 U/mL in N-AGD (*p* = 0.012, Mann–Whitney *U* test) (Table 1).

### 3.4. Histopathology Results

From each AGD patient the histopathological diagnosis was recorded: 13% were normal; 45% had acute rejection; in 30% toxicity was found; and in 13% other diagnosis: acute tubular necrosis, and borderline changes [19].

## 4. Discussion

Renal transplantation is the RRT of choice for patients with ESRD, to provide them with better quality of life and the best survival compared to dialysis, as well as being the most cost-efficient [20]. Besides kidneys from live donors function much better than those from perished individuals. Donors to the AGD group were significantly older (42.54 ± 11.45 years); and although they were found in a state of perfect health, age could be an independent factor in the appearance of AGD. In this regard, it has been previously reported that the acceptable age of live kidney donors continues to be controversial due to the higher incidence of comorbidities and greater risk of postoperative complications [21, 22].

In our study as in our setting, male receptors are 3 : 1 compared to female [10, 23]; this is due to social and epidemiological conditions; perhaps our results show that AGD was most commonly presented in males; all AGD in the period of time were included; nevertheless gender was associated to it presence; due to the fact that all patients have the same immunosuppressive scheme it is possible that this finding was gender-related. Other studies have shown significant correlation in OS in males compared to females although the gender was not a condition to differentiate OS levels (AGD more frequent in men). On the other hand, there is clinical evidence that suggests that male's receptors from female donors could lose graft function [24], and it is well known that male gender is a risk factor for CKD; however, it is necessary to test the hypothesis of the possible association between gender and AGD [25]. In this study, the majority of patients who were subjected to RT were male: 87% of those with AGD and 62% of N-AGD.

One of the primary causes of later loss of the allograft is chronic graft nephropathy, which is characterized, in part, by deterioration of renal function. Registered data demonstrate that renal function in the first posttransplant year is an important predictor of the long-term result in how the RT behaves, where factors that initiate the cycle of the loss of nephrons play a predominant role. In this regard, advanced age, the masculine gender, or diabetes mellitus are considered, among other factors [21, 26].

It has been reported that serum creatinine concentrations of ≤1.5 mg/dL at 6–12 months are associated with greater survival of the graft at 5 years. These serum creatinine results can be predictive of survival as soon as 1 month after transplantation [27]. In patients included in this study AGD was detected between 5 and 8 months after transplant, which suggests that follow-up should be frequent during the first year. It is considered that the restoration of kidney function through RT improves the chronic inflammatory state of the OS associated with uremia, by contributing to improving the survival of patients and the transplanted organ.

The proinflammatory cytokine TNF-*α* is a functional transmembranal homotrimeric of 26-kDa that is liberated in circulation in functional soluble form of 17-kDa. In plasma TNF-*α* is free or it binds with its circulating receptor [28]. The TNF-*α*, in general, is not present in the kidneys. After stimulation by the lipopolysaccharides, the interleukin-1*α*, and during inflammation, the TNF-*α* and its receptors are expressed in the glomerular tuft (endothelial, mesangial, and epithelial) and in the tubular cells [29]. In the patients with AGD the levels of TNF-*α* were not significantly elevated (*p* = 0.64) compared to N-AGD.

The IL-6 in AGD behaved similarly with an increase that was not significant compared to the N-AGD group (*p* = 0.13). The IL-6 is a multifunctional pleiotropic proinflammatory cytokine of 26-kD molecular weight, with the ability to modulate local and systemic immunity [30]. The overproduction of IL-6 leads to the deposit of extracellular matrix proteins, the development of inflammatory lesions, and the synthesis of acute-phase proteins. However, we cannot assume the impact of the overproduction of these proinflammatory cytokines because in the present study their systemic expression was not significant between the groups [31]. With regard to the CRP results, we consider them ambiguous and that the value of the data in relation to the diagnostic precision of the CRP is limited [32]. Although IL-6, TNF-*α*, and CRP are wide studied in clinical studies regarding inflammation, all of them are predictive to mortality and other cardiovascular outcomes in RT patients; up to our knowledge this is the first evaluation of the possible association in AGD of inflammation and OS; however, it is necessary to evaluate other inflammatory markers that may play a role in AGD.

The oxidative state after RT is not entirely known. It is important to consider that oxygen is necessary for the life of aerobic organisms and that the univalent reduction of oxygen leads to the formation of ROS, like the O^2−^. The mechanisms of cellular injury from free radicals involve proteins, lipids, enzymes, receptors, and, at the membrane level, the initiation of lipid peroxidation with the increase in LPO. Lipid peroxidation is put into play as a consequence of the formation of free radicals in the cells and tissues. It is one of the first aspects of abnormal oxidation. The study of the mechanisms of the adverse effects of the LPO identified as aldehydes of the 4-hydroxy-alkenal class [33, 34] is characterized by their high reactivity in the cellular components. In the present study, the LPO were significantly elevated in AGD, which could suggest that the patients with AGD are found in a process of abnormal oxidation.

In 1990, it was demonstrated that the production of a series of F2 compounds of the prostaglandin (known as F2 isoprostanes) that form* in vivo* and* in vitro* by free radicals catalyzes peroxidation of the phospholipid of the arachidonic acid, through an independent pathway of the cyclooxygenase. Although the isoprostanes are less reactive than the other products of peroxidation, they are liberated into circulation, which means that the determination of F2-isoprostanes in serum or plasma could be considered trustworthy markers of OS by lipid peroxidation, to evaluate the oxidative state in diverse human pathologies. The F2 isoprostanes and carbonyl associated protein are elevated in ESRD and descend after RT [17], but there is not any evidence in the AGD setting [14]. In our study, the 8-IP was significantly elevated in AGD, which suggests that 8-IP could induce vasoconstriction effects in the kidney, giving way to alterations in renal function [35].

The endothelial NO synthase (eNOS) is identified as a very important protective factor in renal function [36].

In our study, the levels of NO in AGD were significantly diminished compared to the N-AGD group (*p* = 0.042), in detriment to the endothelial function of the RT, as well as the pathophysiological effects the NO has in relation to OS. The NO is produced from the L-arginine as a result of a catalytic reaction by the NOS enzyme. The NO can control such physiologic processes as important as blood pressure, arterial smooth muscle relaxation, platelet aggregation and adhesion, neurotransmission, and neuroendocrine secretion. It also participates in the destruction of pathogenic microorganisms and tumor cells from leukocytes and macrophages. These functions could be compromised by the diminishing of NO in AGD [37]. The relative scarcity of substance and of cofactors conducts the uncoupling of inducible NOS, resulting in the production of O^2−^ and the activation of transcription factors that later increase the expression of inducible NOS [38].

The diminished activity of the antioxidant enzyme SOD in AGD could mean the persistence of ROS, as was previously reported in a study that found decreased SOD during the early phase of RT that indicated a persistent source of ROS production and of OS. One possible reason could be the TAC-based treatment, since it has been reported that Cyclosporine (another calcineurin inhibitor like TAC) augments the glomerular synthesis of ROS [39].

It is important to consider that TAC remains as the major immunosuppressant drug, and its common adverse effects include arterial hypertension, hyperlipidemia, and hyperglycemia. Pharmacological action of TAC has been previously described; the only known effect of TAC in inflammation is proinflammatory, due to the elevation of ICAM-1 and VCAM-1 dose dependent [40]. This is the reason why TAC cause toxicity. There is no evidence regarding the possible effect of TAC in other inflammatory pathways, although this TAC has been known to decrease OS [41]. Our findings suggest that AGD is a state where OS is elevated and not necessarily due to TAC deficiency or inflammation. It was shown that calcineurin inhibitor exposure induces heat shock protein expression, decreased NO production in cultured tubular epithelial cells, and alterations in calcium influx and free cytosolic calcium concentration, further illustrating the direct toxic effects of calcineurin inhibition on tubular function [42]. Several studies indicate that vascular dysfunction by calcineurin inhibitor results from an increase in vasoconstrictor factors that include endothelin and thromboxane and activation of the renin-angiotensin system, as well as a reduction of vasodilator factors like prostacyclin, prostaglandin E2, and NO [43]. In addition, calcineurin inhibitor induces imbalances in the vasodilator/vasoconstrictor ratio of arachidonic acid metabolites (eicosanoids), which ultimately promotes renal vasoconstriction. The renal vasoconstriction can lead to renal hypoperfusion and hypoxia-reoxygenation injury and subsequently to the formation of ROS or free radicals, which causes cellular injury [44].

Cardiovascular disease is the most common mortality cause after RT; statins have shown a protective lipid lowering effect, but there is no evidence regarding the possible antioxidant effect of statins [45] in the RT setting. Our purpose was not to evaluate the effect of other drugs (instead TAC), that is, statins; it is necessary to design a study* ex profeso*, but there is no evidence regarding the possible pharmacological interaction among TAC and statins in OS [46, 47]. Also we have to consider that AGD is a multifactorial pathological state; OS is only one pathway in its development. To the best of our knowledge, there is no evidence regarding use of antioxidants agents to prevent/treat AGD in RT; due to the observational nature of our results we cannot assure if AGD is cause of consequence of OS; nevertheless a significant association is shown and can be considered as predictors and indicators of AGD. This study shows an opportunity to treat these patients. Our findings indicate that it is possible to consider the evaluation of the effectiveness of antioxidant treatment to prevent and/or treat AGD patients. Clinical studies should be performed to prove this hypothesis; perhaps the choice of antioxidant agent is one of the major challenges, due to pharmacological interactions, immunosuppression treatment, and other medication risks.

In conclusion, we found similar expression of proinflammatory cytokines in patients with and without AGD and deregulation of the OS in patients with AGD.

The limitations of the present study include a small sample size and it was a cross-sectional study. We are conscious that it is convenient to provide close follow-up in patients after modifying the dose of TAC in the first year and periodic long-term follow-up.

## Figures and Tables

**Table 1:** Clinical characteristics, demographics, proinflammatory cytokines, oxidants, and antioxidants. In terms of the recipients, there were significantly more transplantations done in males than females. As a point of inclusion in the study the creatinine was found significantly elevated in AGD, as was urea. The significant older age of the donors could have influenced the AGD. It is attention-grabbing that the inflammatory state between AGD and N-AGD did not predominate. The oxidative state is characterized by significant increases in LPO and 8-IP in AGD and diminished NO and SOD activity.

	N-AGD	AGD	*p*
Demographic and metabolic characteristics			
*Age* (years)	28.08 ± 9.12	25.39 ± 5.71	0.116
*Weight* (kg)	62.21 ± 13.35	69.16 ± 22.37	0.241
*Height* (m)	1.69 ± 0.08	1.66 ± 0.10	0.398
*Gender* F/M, *n* (%)^*∗*^	21/34 (38/62)	7/48 (13/87)	**0.004**
*Glucose* mg/dL	99.57 ± 3.37	100.73 ± 2.24	0.340
*Urea* mg/dL	36.59 ± 1.32	54.78 ± 3.99	**<0.001**
*Cr habitual* (mg/mL)	1.06 ± 0.27	1.19 ± 0.20	**0.002**
*CT* (mg/dL)	152.13 ± 29.16	145.41 ± 35.56	0.230
*LDL* (mg/dL)	77.96 ± 26.66	83.51 ± 25.46	0.214
*HDL* (mg/dL)	43.59 ± 10.82	43.22 ± 5.47	0.474
*VLDL* (mg/dL)	31.11 ± 21.37	35.11 ± 13.52	0.088
*TAG* (mg/dL)	150.41 ± 94.63	161.91 ± 72.78	0.128
*Tobacco* no/yes, *n* (%)^*∗*^	47/8 (85/15)	40/15 (73/27)	0.147
*Alcoholism* no/yes, *n* (%)^*∗*^	46/9 (83/17)	41/14 (76/24)	0.304
*Donor age* (years)	34.07 ± 10.65	42.54 ± 11.45	**<0.001**
*Time after transplant* (months)	8.27 ± 3.78	5.09 ± 3.07	**<0.001**

Proinflammatory cytokines			
*TNF-α* (pg/mL)	125.54 ± 15.92	145.53 ± 18.87	0.636
*IL-6* (pg/mL)	1933.42 ± 235.38	2110.69 ± 350.97	0.129
*CRP* mg/L, *n* (%)^*∗*^			
≤3.19	36 (65.5)	34 (61.5)	0.828
>3.19	19 (34.5)	21 (38.5)	

Oxidants			
*LPO* (*μ*M)	2.41 ± 0.29	4.10 ± 0.69	**0.014**
*8-isoprostanes* (pg/mL)	8.64 ± 1.54	27.47 ± 9.28	**0.012**
*Nitric Oxide* (*μ*mol/L)	190.57 ± 22.04	138.44 ± 19.20	**0.042**

Antioxidants			
*SOD* (U/mL)	11.69 ± 0.55	9.75 ± 0.52	**0.012**

AGD: acute graft dysfunction. Mean ± standard deviation or standard error, *p* = Mann–Whitney *U* test, and ^*∗*^ *p* = Chi^2^ test.
